# Synchronization lag in post stroke: relation to motor function and structural connectivity

**DOI:** 10.1162/netn_a_00105

**Published:** 2019-09-01

**Authors:** Xin Wang, Caio Seguin, Andrew Zalesky, Wan-wa Wong, Winnie Chiu-wing Chu, Raymond Kai-yu Tong

**Affiliations:** Department of Biomedical Engineering, The Chinese University of Hong Kong, Hong Kong, China; Melbourne Neuropsychiatry Centre, Department of Psychiatry, University of Melbourne, Melbourne, Australia; Melbourne Neuropsychiatry Centre, Department of Psychiatry, University of Melbourne, Melbourne, Australia; Department of Biomedical Engineering, University of Melbourne, Melbourne, Australia; Department of Biomedical Engineering, The Chinese University of Hong Kong, Hong Kong, China; Department of Imaging and Interventional Radiology, The Chinese University of Hong Kong, Hong Kong, China; Department of Biomedical Engineering, The Chinese University of Hong Kong, Hong Kong, China

**Keywords:** Functional lag, Chronic stroke, Brain navigation, Network efficiency, Motor function

## Abstract

Stroke is characterized by delays in the resting-state hemodynamic response, resulting in synchronization lag in neural activity between brain regions. However, the structural basis of this lag remains unclear. In this study, we used resting-state functional MRI (rs-fMRI) to characterize synchronization lag profiles between homotopic regions in 15 individuals (14 males, 1 female) with brain lesions consequent to stroke as well as a group of healthy comparison individuals. We tested whether the network communication efficiency of each individual’s structural brain network (connectome) could explain interindividual and interregional variation in synchronization lag profiles. To this end, connectomes were mapped using diffusion MRI data, and communication measures were evaluated under two schemes: shortest paths and navigation. We found that interindividual variation in synchronization lags was inversely associated with communication efficiency under both schemes. Interregional variation in lag was related to navigation efficiency and navigation distance, reflecting its dependence on both distance and structural constraints. Moreover, severity of motor deficits significantly correlated with average synchronization lag in stroke. Our results provide a structural basis for the delay of information transfer between homotopic regions inferred from rs-fMRI and provide insight into the clinical significance of structural-functional relationships in stroke individuals.

## INTRODUCTION

Neuroplasticity refers to the brain’s ability to form new connections throughout life, which is particularly evident in response to traumatic events and stroke. Brain lesions are a long-term consequence of stroke, resulting in deficits in language, motor, or cognition, depending on the lesion location. Through neuroplastic processes, the lesioned brain can reorganize its structure, function, and connections to adapt to such damage (Dimyan & Cohen, 2011; Kolb & Muhammad, 2014). Understanding how brain structure and function reorganize after stroke is crucial in managing stroke recovery.

Resting-state functional MRI (rs-fMRI) has been widely used to characterize brain activity in the absence of external stimuli (Smith et al., 2013). Functional connectivity can be inferred from rs-fMRI data and quantifies the statistical dependence (synchrony) between neural activity at distant brain regions. Previous rs-fMRI studies have delineated intrinsic resting-state networks that can be ascribed to distinct functions, such as vision, executive control, and salience (van den Heuvel & Hulshoff Pol, 2010; Yeo et al., 2011). Regions comprising a common intrinsic network normally exhibit relatively synchronized neural activity. However, synchronization in blood oxygen level-dependent (BOLD) activity between distant (or homotopic) regions comprising the same intrinsic network shows inherent lag due to the time required for signal propagation as well as other neural processes (Mitra, Snyder, Hacker, & Raichle, 2014; Mitra, Snyder, Blazey, & Raichle, 2015). This means that the BOLD activity measured at one region is most strongly synchronized with a delayed, or *temporally shifted* (lagged), version of the BOLD activity at a distant region. These lags in BOLD synchronization can exceed a second in the healthy human brain and may reflect neuronal processes, as opposed to hemodynamic delay (Mitra et al., 2014; Mitra et al., 2018).

Recently, Siegel and colleagues suggested that synchronization lag measured at rest could serve as a potential index of neuroplasticity that indicates behavioral recovery in individuals with stroke (Siegel, Snyder, Ramsey, Shulman, & Corbetta, 2016). Increased lag in BOLD signal synchronization following stroke could be due to two possible reasons. On the one hand, from a local hemodynamic perspective, brain regions share the same vascular supply. Once the shared vascular supply is interrupted because of the effects of cerebral infarction, the hemodynamic response of the intact regions could also be affected, thus leading to increased lag in synchronization (Amemiya, Kunimatsu, Saito, & Ohtomo, 2014; Lv et al., 2013; Siegel, Shulman, & Corbetta, 2017). On the other hand, from a whole-brain perspective, locally damaged tissue may have a brain-wide impact on the structural and functional reorganization of brain networks. Studies have shown that even if structural damage is focal, the function of regions far from the locally damaged site can change after stroke (Carter, Shulman, & Corbetta, 2012; Crofts et al., 2011), including additional recruitment of contralesional motor areas (Lotze et al., 2006; Small, Hlustik, Noll, Genovese, & Solodkin, 2002) and increased activity in the nonprimary motor areas (Tombari et al., 2004; Wong, Chan, Tang, Meng, & Tong, 2013) when performing motor activities. Hence, signal propagation between distant regions may be delayed as a consequence, leading to increased synchronization lag after stroke. This increase in lag may be related to structural-functional reorganization, which has not been extensively investigated in stroke.

Connectomics (Fornito, Zalesky, & Bullmore, 2016) enables quantitative investigation of structural and functional reorganization in individuals with stroke (Caliandro et al., 2017; Laney, Adali, McCombe Waller, & Westlake, 2015; Siegel et al., 2018; Silasi & Murphy, 2014). With this approach, brain connectivity is represented as a graph (network), enabling study of how synchronization in neural activity (i.e., functional connectivity) is facilitated by signal propagation and communication through the brain’s network of white matter pathways. These pathways comprise a sequence of structural connections (white matter fascicles), which can be mapped using diffusion MRI and fiber tracking techniques (Mori, Crain, Chacko, & van Zijl, 1999; Sarwar, Ramamohanarao, & Zalesky, 2019). Signal propagation across the brain’s structural network is conjectured to follow certain communication schemes (Avena-Koenigsberger, Mišić, & Sporns, 2017). A communication scheme dictates the polysynaptic paths that information traverses between pairs of regions that are not directly connected by a white matter fiber. Two widely studied communication schemes are (a) shortest paths (Bullmore & Sporns, 2009; Rubinov & Sporns, 2010) and (b) navigation (Boguñá, Krioukov, & Claffy, 2009; Kleinberg, 2000; Seguin, van den Heuvel, & Zalesky, 2018). Shortest paths traverse the minimal number of synapses (i.e., intermediate nodes) from one region to another. In weighted networks, shortest paths are often determined to minimize the sum of edge weights traversed. The computation of shortest paths mandates assumptions that might be considered unrealistic in biological systems, such as individual regions possessing global knowledge of the full network architecture (Seguin et al., 2018). Navigation relaxes some of these unrealistic assumptions and thereby yields a more biologically plausible model of neural communication than shortest paths. Under navigation, if a region aims to communicate with another region with which it is not directly connected, it establishes a path by progressing to an intermediate region to which it is directly connected. If there are multiple intermediate regions to which it is directly connected, the region that is closest in distance to the desired destination is chosen. Navigation performs particularly well in spatially embedded, scale-free networks like the brain. The feasibility of navigation in brain networks has recently been demonstrated (Allard & Serrano, 2018; Seguin et al., 2018).

Compensatory and recovery mechanisms have been extensively described in the literature, including enhanced axonal growth between areas that are normally not connected in the vicinity of a lesion and long-distance cortico–spinal axonal sprouting (Buchli & Schwab, 2005; Dancause et al., 2005; Wieloch & Nikolich, 2006). Alternative paths that are established to compensate for lesion-induced disruptions in connectivity may traverse longer silent pathways (latent pathways that are inactive) or synapses that are unmasked or activated (Cramer, 2008; Murphy & Corbett, 2009), leading to communication with reduced efficiency and longer delays. In turn, longer delays in communication could potentially manifest as synchronization lag in resting-state functional connectivity. Finding evidence of putative rerouting of neural information flow following stroke can provide new insights into stroke recovery. Furthermore, any potential rerouting of information could be indexed using graph-theoretic measures like communication efficiency and communication distance. These measures may explain interindividual or interregional variation in synchronization lag inferred from rs-fMRI, thereby providing a link between structural connectivity changes and functional lag. We hypothesized that increased synchronization lag would be associated with lower communication efficiency and longer communication distance in the post-stroke connectome.

In the current study, we aim to investigate the structural basis of homotopic synchronization lag in a group of stroke individuals with predominant motor deficits. More specifically, we first aim to test whether interindividual variation in synchronization lag inferred from rs-fMRI can be explained by the efficiency with which information can be communicated through each individual’s post-stroke structural connectome. We then aim to test whether interregional variation in synchronization lag between homotopic regions associates with measures of regional communication efficiency and distance computed in the structural connectome. We thus seek to establish a link between functional alterations and impaired structural communication. Finally, we test whether synchronization lag can predict to severity of motor deficits. Collectively, we aim to develop new insight into recovery mechanisms relating to interregional communication after brain damage due to stroke.

## RESULTS

### Demographic and Clinical Characteristics

Demographic and clinical characteristics of the stroke individuals are listed in Table 1. The stroke patients had moderate-to-severe upper-limb impairment (Action Research Arm Test [ARAT]: 12.62 ± 6.54). More patients had lesions in the right hemisphere (*n* = 10) than in the left hemisphere (*n* = 5), with the majority of infarcts in the territory irrigated by the anterior and middle cerebral arteries. Lesions spanned the following regions: putamen (*n* = 11), insula (*n* = 10), rolandic operculum (*n* = 6), inferior frontal gyrus (*n* = 6), and temporal pole (*n* = 4). There was no significant difference (*p* = 0.165) in age between the stroke (age = 54 ± 8 years) and control (age = 58 ± 3 years) group.

**Table 1.** Demographics and Clinical Characteristics of the Participants

Subject	Age range	Gender	Lesion side	Lesion locations	Stroke type	Stroke onset (y)	ARAT
S1	55–59	M	R	brainstem	I	11	28
S2	60–64	M	L	PLIC, putamen	I	11	14
S3	45–49	M	R	MFG, SFG, precentral, supramarginal, SMA	I	1	19
S4	65–69	M	L	insula, putamen, IFG, temporal pole	H	8	15
S5	65–69	M	R	insula, ITG, IOG, putamen	H	1	12
S6	45–49	M	R	ITG, MTG, STG, MOG, angular, supramarginal	H	0.67	4
S7	60–64	M	R	insula, putamen, rolandic operculum, IFG	I	3	15
S8	55–59	M	L	insula, IFG, putamen	H	5	10
S9	55–59	M	R	insula, IFG, putamen, rolandic operculum, temporal pole	I	7	8
S10	50–54	M	L	putamen, caudate nucleus	I	1	9
S11	40–44	M	R	insula, rolandic operculum, IFG, STG, putamen, temporal pole	H	5	11
S12	40–44	M	R	insula, MTG, STG, putamen, temporal pole, rolandic operculum	H	3	3
S13	55–59	M	R	insula, rolandic operculum, IFG	I	6	16
S14	50–54	F	L	insula, rolandic operculum, putamen	H	3	10
S15	45–49	M	R	insula, putamen	H	1	12
Mean ± SD	54 ± 8					4 ± 3	12 ± 6

*Note*. y = year; M = male; F = female; R = right hemisphere lesion; L = left hemisphere lesion; IFG = inferior frontal gyrus; IOG = inferior occipital gyrus; ITG = inferior temporal gyrus; MFG = middle frontal gyrus; MOG = middle occipital gyrus; MTG = middle temporal gyrus; PLIC = posterior limb of the internal capsule; SFG = superior frontal gyrus; SMA = supplementary motor area; STG = superior temporal gyrus; H = hemorrhagic stroke; I = ischemic stroke; ARAT: Action Research Arm Test (maximum: 57); SD = standard deviation.

### Increased Synchronization Lag in Stroke

**Figure 1.**
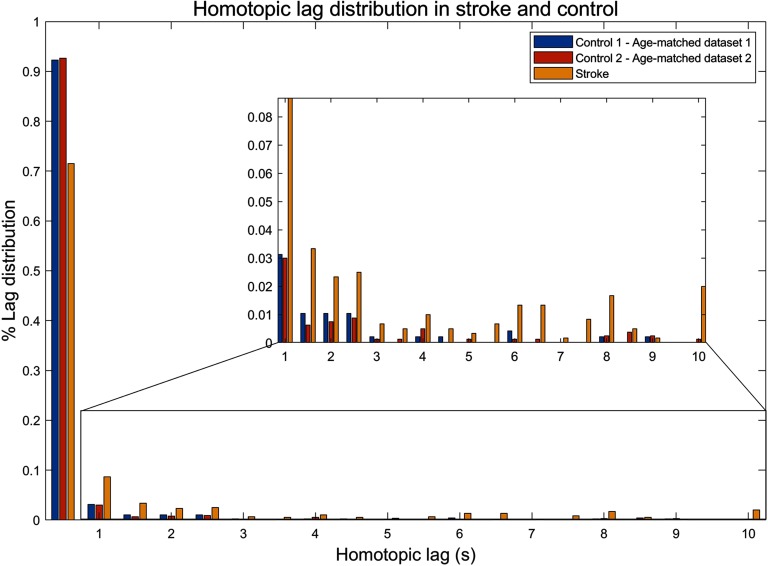
Distribution of synchronization lag in stroke (yellow) and healthy comparison individuals (blue, red) across 40 homotopic regions. The distribution incorporates all 40 pairs of regions and all individuals. Two healthy comparison datasets acquired using different scanners and acquisition protocols were used here. Lag is binned to a temporal resolution of 0.5 seconds. The inset shows the distribution without the lag of zero in order to represent the tail of the distribution more clearly.

Average synchronization lag across 40 homotopic regions was compared between the stroke and healthy comparison group. Lag was significantly increased in the stroke group compared with controls (stroke: mean = 1.07 s, controls: mean = 0.26 s; p < 0.0001, *t* = 5.51). The distribution of synchronization lags (Figure 1) across the 40 homotopic regions suggests a substantially longer tail in the distribution for the stroke individuals, extending to lags of 10 seconds for some regional pairs, whereas lag was typically below a second in the healthy comparison group. Each of the 40 pairs of homotopic regions was tested independently to identify which pairs were associated with the longest increase in lag compared with controls. Among the 40 pairs of homotopic regions, pallidum (*t* = 2.80, *p* = 0.0097, uncorrected) and middle temporal gyrus (*t* = 3.32, *p* = 0.0027, uncorrected) tended to show longer lag than those in the control group. However, none of these 40 pairs of regions survived control of the false discovery rate at 5%.

Considering the relatively small sample size of the control group and the scanner difference, we also tested the lag distribution in two additional healthy comparison groups, including individuals participating in the Human Connectome Project (HCP) (Supporting Information Figure S1). The increased temporal resolution of the HCP data (0.72 s) enabled estimation of lag with greater precision than the clinical data (2 s). We found that the stroke individuals were associated with significantly longer lags when compared with the group of age-matched control (control 2: mean = 0.30 s, *p* < 0.0001, *t* = 6.74) and healthy comparison individuals from the HCP (HCP control: mean = 0.37 s, *p* < 0.0001, *t* = 12.46). These findings are summarized in Table 2. Moreover, we found that synchronization lag did not significantly differ between the three groups of healthy comparison individuals (F = 0.493; p = 0.611). This suggests that interscanner variation is an unlikely explanation for the longer synchronization lags found in the stroke individuals.

**Table 2.** Longer synchronization lag in stroke individuals compared with three independent healthy comparison groups

	Healthy Comparison	Stroke	Healthy vs. Stroke
Control Group 1	0.26 ± 0.12		*t* = 5.5064; *p* = 1.01*e* − 05
Control Group 2	0.30 ± 0.12	1.07 ± 0.49	*t* = 6.7363; *p* = 1.12*e* − 07
HCP	0.37 ± 0.17		*t* = 12.4558; *p* = 4.11*e* − 27

*Note*. HCP = Human Connectome Project.

We next sought to investigate whether stroke-related increases in synchronization lag were global or circumscribed to specific subnetworks. To this end, each of the 40 regional pairs were assigned to one of seven intrinsic resting-state subnetworks delineated in previous studies (Schirner, McIntosh, Jirsa, Deco, & Ritter, 2018; Yeo et al., 2011). The seven subnetworks were as follows: default mode, executive control, sensorimotor, fronto-parietal, auditory, visual, and subcortical networks (Supporting Information). For each individual, each of these seven subnetworks was then classified as either *affected* or *unaffected*, according to whether the subnetwork encompassed a lesion or not, respectively. The set of all affected subnetworks was called the affected network, and the remaining subnetworks were collectively referred to as the unaffected network. In this way, an affected and unaffected network was defined for each individual.

We computed an average synchronization lag for the affected and unaffected network by averaging lags across all pairs of homotopic regions (both lesioned and intact regions) comprising the respective networks. Average synchronization lag was significantly increased in the affected network relative to the unaffected network in the stroke individuals (affected: mean = 1.57 s, unaffected: mean = 0.67 s, *p* = 0.001, *t* = 3.658).

**Figure 2.**
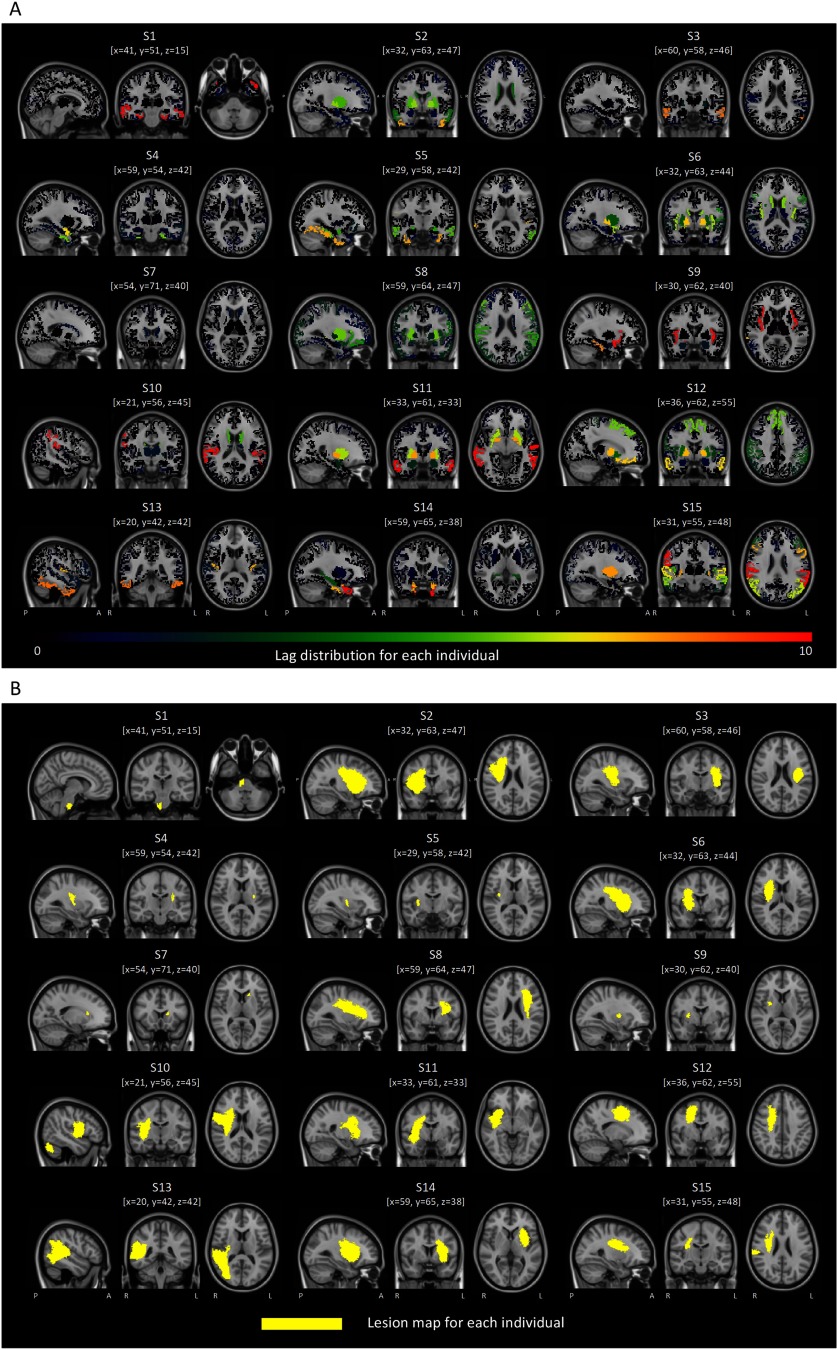
Maps of lag distribution (A) and lesions (B) for each stroke individual. The same brain slice is used in the lag and lesion views, where MNI coordinates are denoted with [x, y, z]. In B, lesions are colored with yellow. Images are shown in MNI152 standard space.

We confirmed that this between-network difference remained significant when considering only intact homotopic pairs belonging to the affected network (*p* = 0.0216, *t* = 2.43). This suggests that lesions may impart downstream effects on unaffected regions within the same network, resulting in lag increases in these unaffected regions. To establish whether this phenomenon was exclusive to the lesioned brain, we performed a control analysis in which the average synchronization lag for the unaffected and affected networks (defined using the stroke data) was computed based on the lag derived from the healthy control group. As expected, no significant difference was found between the two networks in the healthy comparison individuals (*p* > 0.05). This suggests that lag increases are predominantly circumscribed to lesioned regions or regions comprising the same canonical resting-state networks as the lesioned regions. Figure 2 shows the synchronization lag map and the lesion map of each stroke individuals.

### Relation Between Synchronization Lag and Distance to Lesion

**Figure 3.**
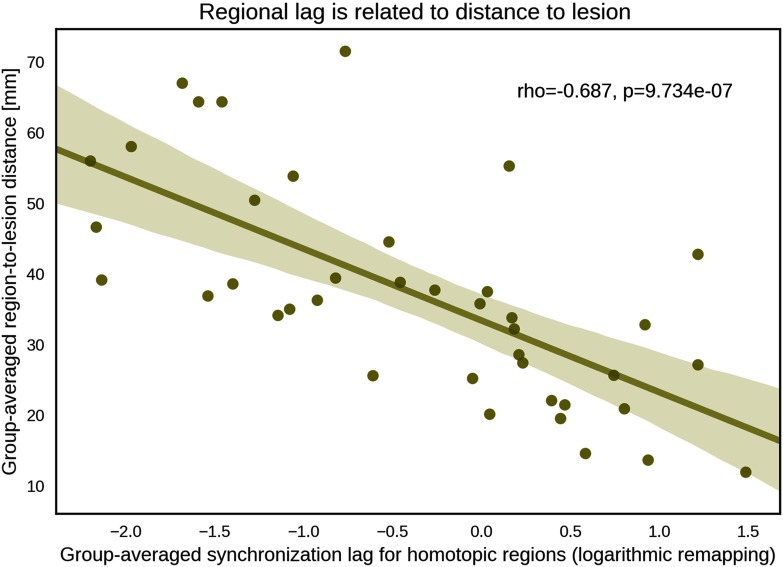
Scatter plot of the negative association between synchronization lag and the Euclidean distance to the lesion. Each data point indicates a homotopic pair of regions. For each pair of homotopic regions, lag was averaged across all stroke individuals and then transformed using a logarithmic remapping. Note that subsecond lags become negative after logarithm transformation. The distance from each pair of homotopic regions to the lesion was defined as the Euclidean distance between the center of mass of the region in the ipsilesional hemisphere and the lesion. In the case of multiple lesions, only the nearest lesion was considered when calculating distance. Distances were also averaged across stroke individuals. ROI = region of interest.

Next, we investigated whether the lag for each pair of homotopic regions was associated with the Euclidean distance between the center of mass of the region in the ipsilesional hemisphere and the lesion. Given the presence of multiple distinct lesions in some individuals, the region-to-lesion distance was computed as the shortest distance between a region and any lesion. Only the regions in the ipsilesional hemisphere were considered when calculating distance. For each pair of homotopic regions, lag and the region-to-lesion distances were averaged across stroke individuals when investigating this relationship. An individual with lesions in the brainstem was excluded, given that this area was not represented in the regional parcellation adopted in this study. We found that the distance to the lesion significantly and negatively correlated with synchronization lag across the 40 homotopic pairs of regions (*r* = −0.687, *p* < 0.0001; Figure 3). To confirm that this relationship was not driven by certain individuals, rather than averaging across individuals and then computing the correlation across regions, we computed an independent correlation across regions for each individual separately. With this approach, we found that the Euclidean distance to the lesion tended to be correlated with synchronization lag in 57% of individuals (Supporting Information Table S1). This result implies that the further a region in the ipsilesional hemisphere is to the lesion, the less the synchronization lag between corresponding pairs of homotopic regions across hemispheres.

### Structural Basis of Synchronization Lag

#### Communication efficiency explains interindividual variation in lag.

We sought to determine a structural basis for the significant increase in synchronization lag that we found in the stroke group. To this end, we first evaluated whether the tractography-derived streamline counts (derived from diffusion MRI data) between homotopic regions related to synchronization lag (derived from functional MRI data). Lag and streamline counts were averaged across homotopic regions for each individual to yield a whole-brain–averaged streamline count and lag for each individual. We found a negative trend toward an association between streamline count and lag across the 40 homotopic regions (*r* = −0.456, *p* = 0.088; Supporting Information Figure S2). Although streamline counts do not have an explicit biological correlate, they are likely to be influenced by axonal myelination, caliber, and end-to-end axonal counts. This suggests that shorter lags tend to be associated with enhanced axonal connectivity between homotopic regions.

**Figure 4.**
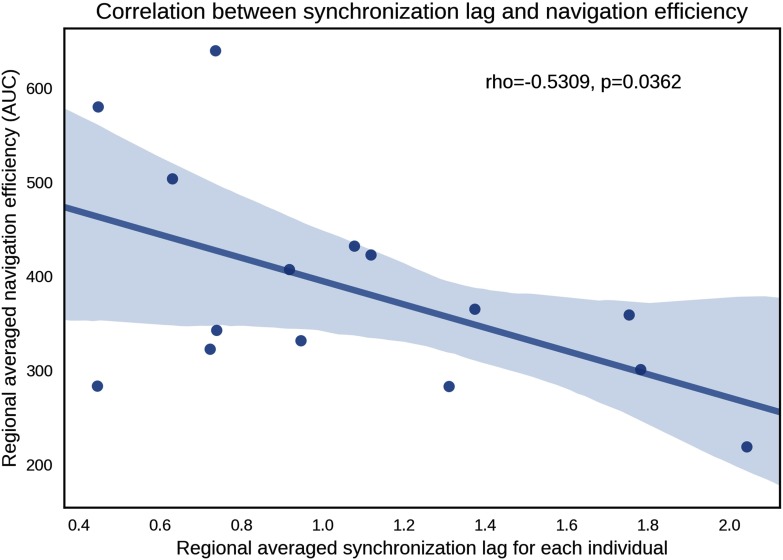
Scatter plot of the association between whole-brain–averaged synchronization lag and navigation efficiency. Each data point represents an individual with stroke. Lags were averaged across all homotopic regions for each individual. Navigation efficiency was summarized using the area under curve (AUC), computed across structural networks with connection density ranging between 10% and 50% at increments of 5%.

To understand the relation between structural measures and lag in greater detail, graph-theoretic analysis of two network communication measures was considered. In particular, we computed network communication efficiency for each stroke individual under two communication schemes: shortest paths and navigation. Whole-brain communication efficiency under these two schemes was computed independently for each stroke individual by using their structural connectome, yielding two efficiency values per individual. For each communication scheme, lags were averaged across all homotopic regions for each individual to yield a whole-brain–averaged measure of shortest paths and navigation efficiency. Lag was found to be significantly correlated with both navigation efficiency (*r* = −0.531, *p* = 0.0362; Figure 4) and shortest path efficiency (*r* = −0.531, *p* = 0.0415). These associations remained significant after controlling for the effects of age and gender. To establish whether this correlation was exclusive to the lesioned brain, we performed the same correlation between efficiency and lag in healthy controls. No significant correlation was found with either navigation efficiency (*r* = −0.093, *p* = 0.568) or shortest path efficiency (*r* = −0.093, *p* = 0.568).

#### Navigation distance explains interregional variation in lag.

We have thus far shown than *interindividual* variation in lag can be explained, to a certain extent, by the efficiency with which information can be communicated in each individual’s structural connectome. We next sought to investigate the structural basis for *interregional* variance in homotopic lag in the stroke individuals. Synchronization lag and efficiency for each pair of homotopic regions were averaged across individuals. We found that synchronization lag was negatively correlated with navigation efficiency across the set of homotopic regions (Spearman rho = −0.3289, *p* = 0.0388), but not for shortest paths efficiency (Spearman rho = −0.1705, *p* = 0.2916). This suggests that the increase in lag evident in the stoke individuals may be due to reduced efficiency of neural signal propagation between homotopic regions.

**Figure 5.**
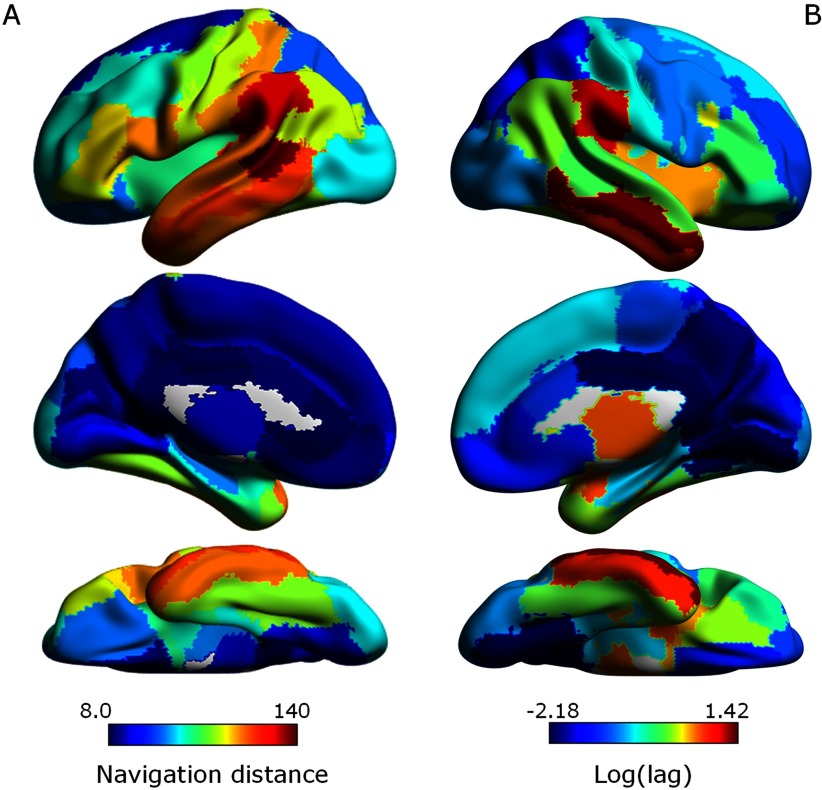
Variation in the efficiency of neural signal propagation between homotopic pairs of regions in the structural connectome associated with interregional variation in synchronization lag. (A) Efficiency of neural signal propagation under navigation between homotopic pairs rendered onto the cortical surface. Efficiency was quantified with the navigation distance. Communication between regions with longer navigation distances (warm colors) is less efficient. (B) Synchronization lag rendered onto the cortical surface following logarithmic remapping. Warm colors indicate longer lags. Note that the two maps tend to have similar distributions.

Following our previous results on the relationship between lag and region-to-lesion distance, we sought to determine whether homotopic Euclidean distance and synchronization lag were associated. Indeed, we found a significant correlation between interregional homotopic distances and lag (*r* = 0.592, *p* < 0.0001), further suggesting the importance of Euclidean distance in shaping synchronization delays. However, neural information transfer is ultimately constrained by underlying white matter pathways, which are not oriented along the straight lines delineated by the Euclidean distance between brain regions. Navigation distance combines anatomical (white matter communication pathways) and geometric (influence of Euclidean distance) constraints into a single information transfer measure (see Methods). Indeed, navigation distance was strongly associated with interregional synchronization lag (*r* = 0.604, *p* < 0.0001; Figure 5). In addition, while Euclidean distance explained 35.08% of the variance in lag, combining navigation and Euclidean distances in a general linear model increased the variance explained to 44.98%. This implies that although Euclidean distance is a significant predictor of lag, synchronization delays are still shaped by underlying network communication facilitated by the structural connectome.

Next, we tested whether the relationship between synchronization lag and navigation/Euclidean distances was present in healthy individuals belonging to the age-matched control group (AMC) and HCP datasets. For both datasets, we found no significant correlation between homotopic lag and navigation distances (AMC: *p* = 0.9868; HCP: *p* = 0.7727), nor between homotopic lag and Euclidean distance (AMC: *p* = 0.2515; HCP: *p* = 0.233). This indicates that the association between increased lag and decrease communication efficiency is specific to the stroke cohort, capturing changes in white matter structure and neural signaling resulting from lesions.

### Relationship Between Lag and Motor Deficit in Stroke

**Figure 6.**
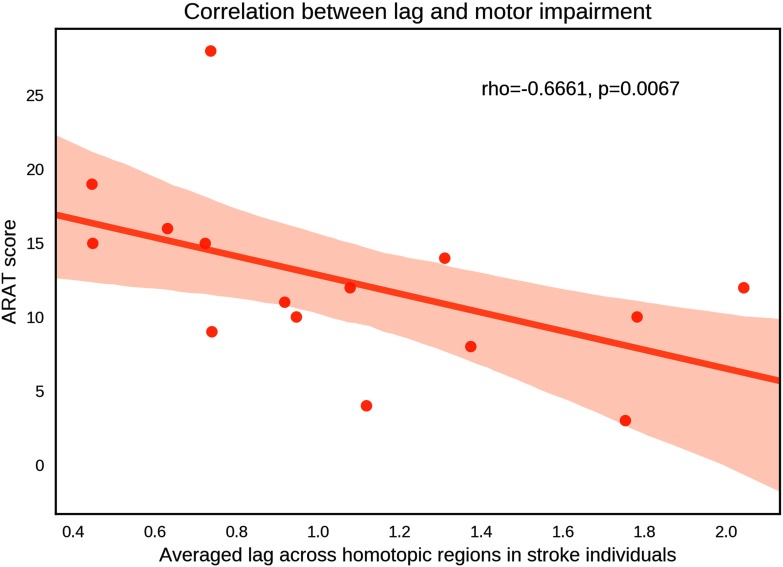
Scatter plot of the association between whole-brain–averaged synchronization lag and motor impairment indicated by Action Research Arm Test (ARAT). Each data point represents an individual with stroke. Lags were averaged across all homotopic regions for each individual.

Finally, we tested whether interindividual variation in average synchronization lag was associated with variation across stroke individuals in motor impairment. ARAT scores quantifying upper-limb motor function were significantly associated with the average lag (*r* = −0.6661, *p* = 0.0067; Figure 6), providing support for the clinical utility of this biomarker. This result indicates that better upper-limb motor function is associated with shorter lag.

We sought to establish whether lag would be a stronger predictor of upper-limb motor impairment compared with the average functional connectivity strength (Pearson correlation) between pairs of homotopic regions. In agreement with previous studies (Carter et al., 2010), although the average functional connectivity was positively correlated with the ARAT scores (*r* = 0.5248, *p* = 0.0447), synchronization lag yielded greater explanatory power and was thus deemed a more sensitive biomarker.

## DISCUSSION

Previous studies suggest that stroke is associated with an increase in synchronization lag between homotopic regions (Siegel et al., 2017, 2016; Zhao, Lambon Ralph, & Halai, 2018). In the present study, we replicated this finding and investigated its structural underpinnings from a graph-theoretic standpoint. We found that both interindividual and interregional variation in stroke-related increases in synchronization lag between homotopic regions could be explained in terms of the efficiency of signal propagation in an individual’s structural connectome. Our findings suggest that lesions may obstruct efficient signaling pathways, requiring recruitment of alternative pathways that traverse longer distances and/or more synapses, resulting in increased propagation delays and synchronization lags. In this way, our results provide a structural basis for the delay of information transfer between homotopic regions in stroke individuals. Moreover, synchronization lag was found to correlate with interindividual motor deficits more strongly than functional connectivity, providing support for the clinical utility of structural and connectomic biomarkers in stroke.

Increased latency in BOLD responses after stroke have been reported in both task-based (Amemiya, Kunimatsu, Saito, & Ohtomo, 2012; Bonakdarpour, Parrish, & Thompson, 2007; Roc et al., 2006) and resting-state (Siegel et al., 2017; Zhao et al., 2018) fMRI studies. We found that synchronization lag was significantly increased in stroke individuals compared with healthy controls, particularly in canonical functional networks encompassing the lesions. These results are in line with previous work demonstrating that functional alterations after stroke extend beyond the area of the lesion (Ovadia-Caro et al., 2013; L. Wang et al., 2010). Regions in the same functional network communicate and interact as a self-organized coalition to complete similar tasks (Sporns, Chialvo, Kaiser, & Hilgetag, 2004). Once part of the network is damaged, lesion-induced functional alterations can also be found in anatomically intact areas that are distant to but connected with the lesion either directly or via a polysynaptic path (Carrera & Tononi, 2014; Laganiere, Boes, & Fox, 2016). These functional alterations may reflect reorganization and/or utilization of redundancy in connectivity to compensate for the lesion-induced network disruptions (Hurtado et al., 2006). Activations occurring in perilesional regions and shifting to other motor areas are commonly observed following stroke with motor deficit (Frías et al., 2018; Grefkes & Fink, 2011). These newly recruited regions cannot fully compensate for the impairment of the lesioned area (Ward & Cohen, 2004), prolonging information transfer and leading to synchronization lag in regions belonging to the same functional network. Accordingly, synchronization lag between homotopic regions increased as a function of the Euclidean distance between the lesion and ipsilesional region. Regions in close spatial proximity to the lesion are likely to share the same vascular supply as the lesion area. Therefore, once a cerebral artery is occluded because of the lesion, vascular supply to surrounding regions may also be comprised. In contrast, regions distant to the lesion are more likely to be served by alternative vascular networks that are minimally influenced by the lesion.

Linking structural and functional properties of the human connectome is a major goal in neuroscience (Amico & Goñi, 2018; Diez et al., 2017; Honey et al., 2009; Mišić et al., 2016; Seguin, Razi, & Zalesky, 2019). In the present study, we focused on understanding the putative link between synchronization lag (functional property) and the efficiency of signal propagation in the connectome (structural property). At both interindividual and interregional levels, signaling efficiency was negatively associated with synchronization lag in the stroke cohort, but not in the healthy control group. Following stroke, synchronization lags emerge most likely because of the obstruction of axonal signaling pathways caused by the lesion and possibly subsequent recruitment of alternative, less efficient pathways. These pathways could be redundant pathways performing similar functions, functionally silent pathways, or sprouting of fibers from the surviving periinfarct neurons (Cramer, 2008; Cramer et al., 2011; Hurtado et al., 2006; Murphy & Corbett, 2009). Compensatory pathways are not necessarily as efficient in communicating information as the original pathways disrupted by the lesion (L. Wang et al., 2010). Our results suggest that graph-theoretic measures of communication efficiency are able to capture aspects of structure-function reorganization following stroke.

Navigation is a decentralized communication scheme that has been proposed as a more biologically plausible model for large-scale neural communication than shortest path routing (Seguin et al., 2018). At the interregional level, navigation efficiency was significantly associated with lag, whereas shortest path efficiency was not. This finding indicates that decentralized models of neural communication may more accurately describe aspects of neural information processing and neuroplasticity (Avena-Koenigsberger et al., 2017). In addition, the geometric nature of neural signaling under the navigation model highlights the importance of both spatial and anatomical constraints to information transfer in the brain.

Interestingly, we also found that the Euclidean distance between homotopic regions was a strong predictor of synchronization lag in stroke. We posit that distant homotopic regions are more vulnerable to disruption because of their use of longer signaling pathways, resulting in an increased likelihood of rerouting. Under navigation, communication pathways are identified based on the distance between regions, while still restricted to the underlying anatomical connections. Hence, measures of communication under navigation capture the three-way relationship between neural signaling, white matter connectivity, and the spatial positioning of regions. Indeed, we found that navigation distance showed the strongest association with synchronization lag out of all measures evaluated, and remained significantly correlated to the lag after controlling for Euclidean distance.

Interhemispheric homotopic functional connectivity is a more accurate predictor of behavioral deficits following stroke than other kinds of functional measures including intrahemispheric connectivity (Carter et al., 2010; Chi et al., 2018; Frías et al., 2018). Consistent with these studies, we also found a significant correlation between homotopic functional connectivity and post-stroke motor deficits. Interestingly, we found that homotopic lag was more strongly associated with motor deficits than functional connectivity. Stroke individuals with better upper-limb motor function tend to have shorter synchronization lag in homotopic regions, possibly suggesting a reorganization of communication between hemispheres facilitating improved function. This is consistent with other studies, indicating that lesions can change communication between the cerebral hemispheres (Jones, 2017; Nowak, Grefkes, Ameli, & Fink, 2009).

Several methodological considerations should be noted. First, image acquisition in the stroke individuals was performed using a different scanner and acquisition sequence than the healthy comparison individuals. This may have resulted in systematic bias when comparing the groups. To assess the impact of this potential bias, we verified our results by using two independent groups of healthy comparison individuals. Consistent with the primary group of healthy comparison individuals, the stroke individuals were still found to show significantly longer synchronization lags when compared with each of these two additional control groups. Furthermore, synchronization lag did not significantly differ between the healthy comparison groups, each of which was acquired using a different scanner and acquisition protocol. This suggests that the estimation of synchronization lag is relatively robust to interscanner differences. Furthermore, our results replicate previous studies showing a significant difference in lag between stroke and healthy comparison individuals (Siegel et al., 2017, 2016). Second, our predominantly male cohort is not representative of the typical sex ratio associated with stroke incidence. We attempted to recruit a balanced sample initially, but then found that males participated in the MRI component of the clinical study at higher rates than females, which resulted in a biased sex ratio. We further note that reanalyzing our data after excluding the single female participant did not alter the significance of the findings reported in this study. Heterogeneity of the sample is another consideration in terms of stroke types and the time since injury. Studies have shown that most functional recovery occurs within the first 3 months after stroke onset (Cramer, 2008; Zeiler & Krakauer, 2013). Given that some of the chronic stroke individuals studied here were still within the recovery window, the possibility of further reorganization in functional connectivity is possible, although the pace of recovery is typically slow (Cramer et al., 2011; Saur et al., 2006) compared with that in the acute stage. Hence, it is possible that ongoing reorganization of functional connectivity could lead to further changes in synchronization lag, which would be evident if follow-up scans were to be acquired. However, synchronization lag was not associated with variation across individuals in the time elapsed from the stroke event to MRI acquisition in the current analysis.

In conclusion, we have proposed and tested a structural explanation for the established phenomenon of synchronization lag in stroke. In particular, we used advanced graph-theoretic analyses of network communication schemes on the connectome to evaluate whether the putative efficiency with which information could be propagated between homotopic regions was related to synchronization lag. This enabled explanation of synchronization lag based on an independent modality (diffusion MRI). We found that interindividual variation in synchronization lag could be explained to a significant extent by network communication efficiency in the post-stroke connectome. Furthermore, interregional variation in lag was associated with navigation distance, which is dependent on both Euclidean distance and structural constraints. Finally, the correlation between synchronization lag and motor deficits provided support for the clinical utility of this biomarker in stroke recovery.

## METHODS

### Participants

Fifteen individuals with chronic stroke (14 males and 1 female; mean age: 54 ± 8 years) were selected from an ongoing clinical motor training trial, which is described in detail elsewhere (NCT03286309). All individuals who participated in this trial and completed an MRI scan were included. All individuals suffered from a single cerebrovascular accident prior to recruitment. The inclusion criteria were (a) sufficient cognition to follow experimental instructions, determined by a Mini-Mental State Examination (MMSE) score exceeding 21, and (b) hemiparesis resulting from a single unilateral brain lesion with stroke onset more than 6 months before recruitment. Exclusion criteria were (a) history of alcohol, drug abuse, or epilepsy, and (b) bilateral infracts, uncontrolled medical problems, and serious cognitive deficits. Motor deficits were the primary stroke-related symptom in all individuals. Compared with previous studies of synchronization lag in stroke (Siegel et al., 2017, 2016), our cohort is relatively homogeneous with respect to clinical symptoms, in that it is constrained to individuals with motor deficits. Paretic upper-limb motor functions were assessed by trained clinical assessors who were blinded to the experiment. Action Research Arm Test (ARAT) was used to evaluate the paretic upper-limb motor function. Twelve right-handed and age-matched healthy comparison individuals (6 males and 6 females; mean age: 58 ± 3 years) without any cerebral abnormalities were included to serve as a control group. The control group was sourced from the open database of Institute of Psychology, Chinese Academy of Sciences (G.-X. Wei, Dong, Yang, Luo, & Zuo, 2014). All individuals gave informed consent of the study protocol. The study was approved by the Joint Chinese University of Hong Kong-New Territories East Cluster Clinical Research Ethics Committee.

### Image Acquisition

Stroke individuals were scanned with a 3T Philips MR scanner (Achieva TX, Philips Medical System, Best, the Netherlands) with an 8-channel head coil. The imaging datasets of healthy controls were acquired from an open database (https://doi.org/10.15387/fcp_indi.corr.ipcas8), where scanning was performed with 3T MRI scanner (Siemens Trio Tim, Erlangen, Germany) with a 12-channel head coil. The following imaging data were acquired in both groups: (a) high-resolution T1-weighted anatomical images, (b) BOLD fMRI images, and (c) diffusion-weighted images. The detailed acquisition parameters can be found in the Supporting Information. The resting-state fMRI acquisition lasted for 8 minutes resulting in 240 brain volumes for both stroke and control scans.

Given that the healthy comparison individuals were scanned on a different scanner to the stroke individuals, we tested for potential interscanner differences in synchronization lag estimation. To this end, two alternative groups of healthy comparison individuals were analyzed: (a) 20 age-matched control subjects from an open dataset (D. Wei et al., 2018) and (b) 200 healthy individuals from the HCP (Van Essen et al., 2013). The detailed acquisition parameters can be found elsewhere (Glasser et al., 2013; Seguin et al., 2018) and in the Supporting Information. In total, we therefore evaluated synchronization lags in three independent groups of healthy comparison individuals. Each of the three groups was acquired using different scanners and acquisition protocols.

### Data Preprocessing and Analysis

#### Functional MRI data preprocessing.

The fMRI data were preprocessed using Analysis of Functional NeuroImages (AFNI) software (http://afni.nimh.nih.gov/afni). The analysis steps followed the recommended analysis procedures for rs-fMRI data (Jo et al., 2013; Jo, Saad, Simmons, Milbury, & Cox, 2010). The first 10 volumes of each individual’s fMRI data were removed. After preprocessing, our procedures included despiking of large transients, slice-timing correction, and motion correction with six-parameter rigid body transformation. Volumes were spatially filtered by a 4-mm full-width-at-half-maximum isotropic Gaussian kernel. T1 images were segmented into gray matter, white matter, and CSF by using FreeSurfer (Fischl et al., 2002) for each individual to create corresponding masks. The time series from lateral ventricles and white matter were then derived for nuisance regression. Other nuisances included motion parameters and motion parameter time derivatives. Volumes with excessive motion were censored if the Euclidean norm of the derivatives of the motion parameters exceeded 0.2 by using the function *regress_censor_motion* in AFNI (X. Wang, Wong, Sun, Chu, & Tong, 2018). Bandpass filtering (0.009–0.08 Hz) was also applied. Global signal regression was not used in the processing.

#### Diffusion data processing and fiber tractography.

Diffusion-weighted images were first preprocessed using the FMRIB Software Library, including correction for eddy currents and brain extraction. Structural networks (connectomes) were then mapped for each individual using MRtrix3 software (http://www.mrtrix.org/). White matter masks for fiber tracking were derived from FreeSurfer-based structural segmentations. Diffusion tensors were estimated from the preprocessed diffusion images using an iteratively reweighted linear least squares estimator. To avoid the potential gaps between gray and white matter due to registration error, the white matter boundaries were dilated by 1 voxel. Streamlines were uniformly seeded from the white matter mask and propagated according to the principal orientation of the estimated diffusion tensor. Propagation was terminated when streamlines exited the white matter mask into gray matter or reached a voxel with low fractional anisotropy (FA) (Seguin et al., 2018). Deterministic diffusion fiber tracking was performed using the FACT tracking algorithm with 5 × 10^6^ streamlines, 0.5-mm tracking step-size, 400-mm maximum streamline length, and 0.1 FA cutoff for termination of tracks. We used deterministic tracking rather than probabilistic to conform to recent connectome mapping recommendations (Maier-Hein et al., 2017; Sarwar et al., 2019). The connectivity strength between a pair of gray matter regions was defined as the total number of streamlines with endpoints residing in both regions. For a given parcellation comprising N gray matter regions, this resulted in an N × N weighted connectivity matrix for each individual.

### Measurement of Synchronization Lag

Cortical gray matter was parcellated into *N* = 80 regions of interest (40 regions per hemisphere) based on the Desikan/Killiany atlas (Desikan et al., 2006). In keeping with previous studies (Siegel et al., 2017), synchronization lag was only quantified between pairs of homotopic regions, primarily because functional connectivity was greatest between homotopic regions in the healthy comparison individuals, thus enabling accurate estimation of cross-correlations. Furthermore, interhemispheric functional connectivity is typically investigated in stroke since it shows a relationship between ipsilesional and contralesional hemisphere and has previously been associated with deficits after stroke (Carter et al., 2010). The synchronization lag in the hemodynamic response between a pair of homotopic regions was derived from the cross-correlation in regionally-averaged BOLD time series signals (Mitra et al., 2014; Siegel et al., 2016):

$${\text{C}}_{\text{i,j}}(\tau )=\frac{1}{n_{\tau }}\underset{t}{\sum }s_{i}(t)\cdot s_{j}(t+\tau )$$ (1)

where s_i_ and s_j_ are the de-meaned and standard deviation normalized BOLD time series signals of homotopic regions i and j. Cross-correlation was computed separately for temporal shifts (*τ*) between −10 and 10 seconds, corresponding to shifts in TR between −5 and 5. In the above formula, *n*_*τ*_ denotes the number of time points comprising the signal with temporal shift of *τ*. The synchronization lag for regions i and j was then defined as,

$$\tau_{ij}=\text{arg}\underset{\tau \in \left[-10,10\right]}{\max}\left|C_{ij}(\tau )\right|$$ (2)

In words, the synchronization lag is the temporal shift (lag), denoted with *τ*, yielding maximal cross-correlation in BOLD activity. Given the relatively coarse temporal resolution of the BOLD signal, parabolic interpolation between consecutive time points was used to determine the exact lag value resulting in maximal cross-correlation. Therefore, each pair of homotopic regions for each individual was associated with a synchronization lag. In some analyses, lag was averaged across all homotopic regions to yield an average synchronization lag for each individual.

### Network Communication Measures

We computed network communication efficiency under two communication schemes: shortest path and navigation. Let *W* denote a weighted connectivity matrix, where *W*_*ij*_ is the connection weight (streamline count) between nodes i and j. We computed the connection length matrix *L* = −log_10_(*W*/max(*W*) + 1), ensuring a monotonic remapping of connection weights into connection lengths that allows for the computation of communication path lengths (Goñi et al., 2014; Seguin et al., 2018). Path lengths were defined as Λ_*ij*_ = *L*_*iu*_ + ⋯ + *L*_*vj*_, where {u … *v*} is the sequence of nodes comprised along the shortest or navigation paths from node i to node j. The shortest path length $\Lambda_{ij}^{sp}$ is the globally minimum communication cost between nodes i and j. In contrast, navigation path length $\Lambda_{ij}^{nav}$ expresses the cost of communication paths identified using local knowledge of network topology and the spatial positioning of nodes. Navigation paths from node i to j were identified by progressing to the neighbor of i closest in Euclidean distance to j. This process was repeated for each new node until j is reached (successful navigation) or a node is revisited (failed navigation, resulting in $\Lambda_{ij}^{nav}$ = ∞).

The communication cost of navigation can be alternatively defined in terms of the Euclidean distance traversed along navigation paths. We refer to this measure as navigation distance and define it as $D_{ij}^{nav}$ = *d*_*iu*_ + ⋯ + *d*_*vj*_, where *d*_*ij*_ is the Euclidean distance between noedes i and j, and {u, …, v} is the sequence of node along the navigation path.

The network efficiency of the brain was computed based on corresponding path length:

$$E=\frac{\sum_{i\neq j}{\left(\Lambda_{ij}\right)}^{-1}}{N\left(N-1\right)}$$ (3)

where *N* was the number of regions in brain parcellation. All the graph-theoretical analyses were computed using the Brain Connectivity Toolbox (https://sites.google.com/site/bctnet/) (Rubinov & Sporns, 2010).

### Statistics

Statistical inference was conducted using MATLAB (The MathWorks). Two-sample, two-sided *t* tests were used to test the null hypothesis of equality in lag between the stroke and healthy comparison group. Each of the 40 pairs of homotopic regions was tested independently as well. To evaluate the impact of structural communication measures on synchronization lag, the Pearson correlation coefficient was computed between mean synchronization lag and measures of the structural network. Network efficiency was summarized using the AUC, computed across structural networks with connection density ranging between 10% and 50% at 5% intervals. For interregional analysis, all the lag values were transformed using logarithm given that the lag distribution in homotopic regions was not normally distributed. The relationship between lag and stroke motor function was tested with the Spearman correlation coefficient. The statistical significance level was set at *p* < 0.05. All *p* values were corrected using the false discovery rate.

## ACKNOWLEDGMENTS

We thank the Research Grants Council of the Hong Kong Special Administrative Region and CUHK Global Scholarship Programme for Research Excellence.

## SUPPORTING INFORMATION

Supporting information for this article is available at https://www.doi.org/10.1162/netn_a_00105.

## AUTHOR CONTRIBUTIONS

Xin Wang: Data curation; Formal analysis; Methodology; Validation; Visualization; Writing – Original Draft; Writing – Review & Editing. Caio Seguin: Data curation; Formal analysis; Methodology; Writing – Original Draft; Writing – Review & Editing. Andrew Zalesky: Conceptualization; Data curation; Formal analysis; Funding acquisition; Investigation; Methodology; Supervision; Writing – Original Draft; Writing – Review & Editing. Wan-wa Wong: Data curation; Formal analysis; Methodology; Writing – Original Draft; Writing – Review & Editing. Winnie Chiu-wing Chu: Conceptualization; Project administration; Software; Writing – Original Draft. Raymond Kai-yu Tong: Conceptualization; Funding acquisition; Project administration; Supervision; Writing – Original Draft.

## FUNDING INFORMATION

Raymond Kai-yu Tong, Research Grants Council of the Hong Kong Special Administrative Region, Award ID: GRF-CUHK 14207617. Xin Wang, CUHK Global Scholarship Programme for Research Excellence. Andrew Zalesky, Australian National Health and Medical Research Council (NHMRC) Senior Research Fellowship B, Award ID: 1136649. Caio Seguin, Melbourne Research Scholarship.

## TECHNICAL TERMS

synchronization lag: The temporal delay between the fMRI signals measured at two distinct brain regions, typically computed using the cross-correlation function.
navigation: A network communication model describing how information is routed across multi-hop (polysynaptic) paths in a network.
spatially-embedded network: A network with an inherent spatial layout and/or geometric attributes, contrast to networks that are purely topological.
scale-free network: A network where the distribution of the node degree follows a power law, containing hub nodes with a degree that substantially exceeds the average degree in the network.
homotopic regions: The same regions but in different hemispheres. Inter-hemispheric functional connectivity is typically stronger between mirror (homotopic) regions of the brain.
ipsilesional hemisphere: The brain hemisphere that is on the same side as a lesion.
navigation distance: The length of a communication path between two nodes quantified under navigation.
decentralized communication: A network communication model that does not mandate global knowledge of the network topology to determine communication paths, contrast to centralized communication models.
connection density: The proportion of actual connected edges among all possible connections in a network.

## References

[bib1] Allard A., & Serrano M. A. (2018). Navigable maps of structural brain networks across species. ArXiv:1801.06079 [Physics, q-Bio]. Retrieved from http://arxiv.org/abs/180 1.06079 10.1371/journal.pcbi.1007584 PMC7018228 32012151

[bib2] Amemiya S., Kunimatsu A., Saito N., & Ohtomo K. (2012). Impaired hemodynamic response in the ischemic brain assessed with BOLD fMRI. NeuroImage, 61(3), 579–590. 10.1016/j.neuroimage.2012.04.001 22507231

[bib3] Amemiya S., Kunimatsu A., Saito N., & Ohtomo K. (2014). Cerebral hemodynamic impairment: assessment with resting-state functional MR imaging. Radiology, 270(2), 548–555. 10.1148/radiol.13130982 24072777

[bib4] Amico E., & Goñi J. (2018). Mapping hybrid functional-structural connectivity traits in the human connectome. Network Neuroscience, 2(3), 306–322. 10.1162/netn_a_00049 30259007 PMC6145853

[bib5] Avena-Koenigsberger A., Mišić B., & Sporns O. (2017). Communication dynamics in complex brain networks. Nature Reviews Neuroscience, 19, 17. 29238085 10.1038/nrn.2017.149

[bib6] Boguñá M., Krioukov D., & Claffy K. C. (2009). Navigability of complex networks. Nature Physics, 5(1), 74–80. 10.1038/nphys1130

[bib7] Bonakdarpour B., Parrish T. B., & Thompson C. K. (2007). Hemodynamic response function in patients with stroke-induced aphasia: implications for fMRI data analysis. NeuroImage, 36(2), 322–331. 10.1016/j.neuroimage.2007.02.035 17467297 PMC2041913

[bib8] Buchli A. D., & Schwab M. E. (2005). Inhibition of Nogo: a key strategy to increase regeneration, plasticity and functional recovery of the lesioned central nervous system. Annals of Medicine, 37(8), 556–567. 10.1080/07853890500407520 16338758

[bib9] Bullmore E., & Sporns O. (2009). Complex brain networks: graph theoretical analysis of structural and functional systems. Nature Reviews Neuroscience, 10(3), 186–198. 10.1038/nrn2575 19190637

[bib10] Caliandro P., Vecchio F., Miraglia F., Reale G., Della Marca G., La Torre G., … Rossini P. M. (2017). Small-world characteristics of cortical connectivity changes in acute stroke. Neurorehabilitation and Neural Repair, 31(1), 81–94. 10.1177/1545968316662525 27511048

[bib11] Carrera E., & Tononi G. (2014). Diaschisis: past, present, future. Brain: A Journal of Neurology, 137(Pt 9), 2408–2422. 10.1093/brain/awu101 24871646

[bib12] Carter A. R., Astafiev S. V., Lang C. E., Connor L. T., Rengachary J., Strube M. J., … Corbetta M. (2010). Resting interhemispheric functional magnetic resonance imaging connectivity predicts performance after stroke. Annals of Neurology, 67(3), 365–375. 10.1002/ana.21905 20373348 PMC2927671

[bib13] Carter A. R., Shulman G. L., & Corbetta M. (2012). Why use a connectivity-based approach to study stroke and recovery of function?. NeuroImage, 62(4), 2271–2280. 10.1016/j.neuroimage.2012.02.070 22414990 PMC3733251

[bib14] Chi N.-F., Ku H.-L., Chen D. Y.-T., Tseng Y.-C., Chen C.-J., Lin Y.-C., … Hu C.-J. (2018). Cerebral motor functional connectivity at the acute stage: an outcome predictor of ischemic stroke. Scientific Reports, 8(1). 10.1038/s41598-018-35192-y PMC6235876 30429535

[bib15] Cramer S. C. (2008). Repairing the human brain after stroke: I. Mechanisms of spontaneous recovery. Annals of Neurology, 63(3), 272–287. 10.1002/ana.21393 18383072

[bib16] Cramer S. C., Sur M., Dobkin B. H., O’Brien C., Sanger T. D., Trojanowski J. Q., … Vinogradov S. (2011). Harnessing neuroplasticity for clinical applications. Brain: A Journal of Neurology, 134(Pt 6), 1591–1609. 10.1093/brain/awr039 21482550 PMC3102236

[bib17] Crofts J. J., Higham D. J., Bosnell R., Jbabdi S., Matthews P. M., Behrens T. E. J., & Johansen-Berg H. (2011). Network analysis detects changes in the contralesional hemisphere following stroke. NeuroImage, 54(1), 161–169. 10.1016/j.neuroimage.2010.08.032 20728543 PMC3677803

[bib18] Dancause N., Barbay S., Frost S. B., Plautz E. J., Chen D., Zoubina E. V., … Nudo R. J. (2005). Extensive cortical rewiring after brain injury. Journal of Neuroscience, 25(44), 10167–10179. 10.1523/JNEUROSCI.3256-05.2005 16267224 PMC6725801

[bib19] Desikan R. S., Ségonne F., Fischl B., Quinn B. T., Dickerson B. C., Blacker D., … Killiany R. J. (2006). An automated labeling system for subdividing the human cerebral cortex on MRI scans into gyral based regions of interest. NeuroImage, 31(3), 968–980. 10.1016/j.neuroimage.2006.01.021 16530430

[bib20] Diez I., Drijkoningen D., Stramaglia S., Bonifazi P., Marinazzo D., Gooijers J., … Cortes J. M. (2017). Enhanced prefrontal functional–structural networks to support postural control deficits after traumatic brain injury in a pediatric population. Network Neuroscience, 1(2), 116–142. 10.1162/NETN_a_00007 29911675 PMC5988395

[bib21] Dimyan M. A., & Cohen L. G. (2011). Neuroplasticity in the context of motor rehabilitation after stroke. Nature Reviews Neurology, 7(2), 76–85. 10.1038/nrneurol.2010.200 21243015 PMC4886719

[bib22] Fischl B., Salat D. H., Busa E., Albert M., Dieterich M., Haselgrove C., … Dale A. M. (2002). Whole brain segmentation: automated labeling of neuroanatomical structures in the human brain. Neuron, 33(3), 341–355. 11832223 10.1016/s0896-6273(02)00569-x

[bib23] Fornito A., Zalesky A., & Bullmore E. T. (2016). Fundamentals of brain network analysis. Boston: Elsevier/Academic Press.

[bib24] Frías I., Starrs F., Gisiger T., Minuk J., Thiel A., & Paquette C. (2018). Interhemispheric connectivity of primary sensory cortex is associated with motor impairment after stroke. Scientific Reports, 8(1). 10.1038/s41598-018-29751-6 PMC6105621 30135496

[bib25] Glasser M. F., Sotiropoulos S. N., Wilson J. A., Coalson T. S., Fischl B., Andersson J. L., … WU-Minn HCP Consortium (2013). The minimal preprocessing pipelines for the Human Connectome Project. NeuroImage, 80, 105–124. 10.1016/j.neuroimage.2013.04.127 23668970 PMC3720813

[bib26] Goñi J., van den Heuvel M. P., Avena-Koenigsberger A., Velez de Mendizabal N., Betzel R. F., Griffa A., … Sporns O. (2014). Resting-brain functional connectivity predicted by analytic measures of network communication. Proceedings of the National Academy of Sciences of the United States of America, 111(2), 833–838. 10.1073/pnas.1315529111 24379387 PMC3896172

[bib27] Grefkes C., & Fink G. R. (2011). Reorganization of cerebral networks after stroke: new insights from neuroimaging with connectivity approaches. Brain: A Journal of Neurology, 134(Pt 5), 1264–1276. 10.1093/brain/awr033 21414995 PMC3097886

[bib28] Honey C. J., Sporns O., Cammoun L., Gigandet X., Thiran J. P., Meuli R., & Hagmann P. (2009). Predicting human resting-state functional connectivity from structural connectivity. Proceedings of the National Academy of Sciences of the United States of America, 106(6), 2035–2040. 10.1073/pnas.0811168106 19188601 PMC2634800

[bib29] Hurtado O., Pradillo J. M., Alonso-Escolano D., Lorenzo P., Sobrino T., Castillo J., … Moro M. A. (2006). Neurorepair versus neuroprotection in stroke. Cerebrovascular Diseases (Basel, Switzerland), 21 Suppl 2, 54–63. 10.1159/000091704 16651815

[bib30] Jo H. J., Gotts S. J., Reynolds R. C., Bandettini P. A., Martin A., Cox R. W., & Saad Z. S. (2013). Effective preprocessing procedures virtually eliminate distance-dependent motion artifacts in resting state FMRI. Journal of Applied Mathematics, 2013, 1–9. 10.1155/2013/935154 PMC3886863 24415902

[bib31] Jo H. J., Saad Z. S., Simmons W. K., Milbury L. A., & Cox R. W. (2010). Mapping sources of correlation in resting state fMRI, with artifact detection and removal. NeuroImage, 52(2), 571–582. 10.1016/j.neuroimage.2010.04.246 20420926 PMC2897154

[bib32] Jones T. A. (2017). Motor compensation and its effects on neural reorganization after stroke. Nature Reviews Neuroscience, 18(5), 267–280. 10.1038/nrn.2017.26 28331232 PMC6289262

[bib33] Kleinberg J. M. (2000). Navigation in a small world. Nature, 406, 845. 10972276 10.1038/35022643

[bib34] Kolb B., & Muhammad A. (2014). Harnessing the power of neuroplasticity for intervention. Frontiers in Human Neuroscience, 8, 377 10.3389/fnhum.2014.00377 25018713 PMC4072970

[bib35] Laganiere S., Boes A. D., & Fox M. D. (2016). Network localization of hemichorea-hemiballismus. Neurology, 86(23), 2187–2195. 10.1212/WNL.0000000000002741 27170566 PMC4898318

[bib36] Laney J., Adali T., McCombe Waller S., & Westlake K. P. (2015). Quantifying motor recovery after stroke using independent vector analysis and graph-theoretical analysis. NeuroImage Clinical, 8, 298–304. 10.1016/j.nicl.2015.04.014 26106554 PMC4474175

[bib37] Lotze M., Markert J., Sauseng P., Hoppe J., Plewnia C., & Gerloff C. (2006). The role of multiple contralesional motor areas for complex hand movements after internal capsular lesion. Journal of Neuroscience, 26(22), 6096–6102. 10.1523/JNEUROSCI.4564-05.2006 16738254 PMC6675223

[bib38] Lv Y., Margulies D. S., Cameron Craddock R., Long X., Winter B., Gierhake D., … Villringer A. (2013). Identifying the perfusion deficit in acute stroke with resting-state functional magnetic resonance imaging. Annals of Neurology, 73(1), 136–140. 10.1002/ana.23763 23378326

[bib39] Maier-Hein K. H., Neher P. F., Houde J.-C., Côté M.-A., Garyfallidis E., Zhong J., … Descoteaux M. (2017). The challenge of mapping the human connectome based on diffusion tractography. Nature Communications, 8(1). 10.1038/s41467-017-01285-x PMC5677006 29116093

[bib40] Mišić B., Betzel R. F., de Reus M. A., van den Heuvel M. P., Berman M. G., McIntosh A. R., & Sporns O. (2016). Network-level structure-function relationships in human neocortex. Cerebral Cortex, 26(7), 3285–3296. 10.1093/cercor/bhw089 27102654 PMC4898678

[bib41] Mitra A., Kraft A., Wright P., Acland B., Snyder A. Z., Rosenthal Z., … Raichle M. E. (2018). Spontaneous infra-slow brain activity has unique spatiotemporal dynamics and laminar structure. Neuron, 98(2), 297–305.e6. 10.1016/j.neuron.2018.03.015 29606579 PMC5910292

[bib42] Mitra A., Snyder A. Z., Blazey T., & Raichle M. E. (2015). Lag threads organize the brain’s intrinsic activity. Proceedings of the National Academy of Sciences of the United States of America, 112(17), E2235–E2244. 10.1073/pnas.1503960112 25825720 PMC4418865

[bib43] Mitra A., Snyder A. Z., Hacker C. D., & Raichle M. E. (2014). Lag structure in resting-state fMRI. Journal of Neurophysiology, 111(11), 2374–2391. 10.1152/jn.00804.2013 24598530 PMC4097876

[bib44] Mori S., Crain B. J., Chacko V. P., & van Zijl P. C. (1999). Three-dimensional tracking of axonal projections in the brain by magnetic resonance imaging. Annals of Neurology, 45(2), 265–269. 9989633 10.1002/1531-8249(199902)45:2<265::aid-ana21>3.0.co;2-3

[bib45] Murphy T. H., & Corbett D. (2009). Plasticity during stroke recovery: from synapse to behaviour. Nature Reviews Neuroscience, 10(12), 861–872. 10.1038/nrn2735 19888284

[bib46] Nowak D. A., Grefkes C., Ameli M., & Fink G. R. (2009). Interhemispheric competition after stroke: brain stimulation to enhance recovery of function of the affected hand. Neurorehabilitation and Neural Repair, 23(7), 641–656. 10.1177/1545968309336661 19531606

[bib47] Ovadia-Caro S., Villringer K., Fiebach J., Jungehulsing G. J., van der Meer E., Margulies D. S., & Villringer A. (2013). Longitudinal effects of lesions on functional networks after stroke. Journal of Cerebral Blood Flow and Metabolism, 33(8), 1279–1285. 10.1038/jcbfm.2013.80 23715061 PMC3734780

[bib48] Roc A. C., Wang J., Ances B. M., Liebeskind D. S., Kasner S. E., & Detre J. A. (2006). Altered hemodynamics and regional cerebral blood flow in patients with hemodynamically significant stenoses. Stroke, 37(2), 382–387. 10.1161/01.STR.0000198807.31299.43 16373653

[bib49] Rubinov M., & Sporns O. (2010). Complex network measures of brain connectivity: uses and interpretations. NeuroImage, 52(3), 1059–1069. 10.1016/j.neuroimage.2009.10.003 19819337

[bib50] Sarwar T., Ramamohanarao K., & Zalesky A. (2019). Mapping connectomes with diffusion MRI: deterministic or probabilistic tractography? Magnetic Resonance in Medicine, 81(2), 1368–1384. 10.1002/mrm.27471 30303550

[bib51] Saur D., Lange R., Baumgaertner A., Schraknepper V., Willmes K., Rijntjes M., & Weiller C. (2006). Dynamics of language reorganization after stroke. Brain: A Journal of Neurology, 129(Pt 6), 1371–1384. 10.1093/brain/awl090 16638796

[bib52] Schirner M., McIntosh A. R., Jirsa V., Deco G., & Ritter P. (2018). Inferring multi-scale neural mechanisms with brain network modelling. ELife, 7 10.7554/eLife.28927 PMC5802851 29308767

[bib53] Seguin C., Razi A., & Zalesky A. (2019). Inferring neural signalling directionality from undirected structural connectomes. BioRxiv. 10.1101/573071 PMC6753104 31537787

[bib54] Seguin C., van den Heuvel M. P., & Zalesky A. (2018). Navigation of brain networks. Proceedings of the National Academy of Sciences of the United States of America, 115(24), 6297–6302. 10.1073/pnas.1801351115 29848631 PMC6004443

[bib55] Siegel J. S., Seitzman B. A., Ramsey L. E., Ortega M., Gordon E. M., Dosenbach N. U. F., … Corbetta M. (2018). Re-emergence of modular brain networks in stroke recovery. Cortex, 101, 44–59. 10.1016/j.cortex.2017.12.019 29414460 PMC6527102

[bib56] Siegel J. S., Shulman G. L., & Corbetta M. (2017). Measuring functional connectivity in stroke: Approaches and considerations. Journal of Cerebral Blood Flow and Metabolism, 37(8), 2665–2678. 10.1177/0271678x17709198 28541130 PMC5536814

[bib57] Siegel J. S., Snyder A. Z., Ramsey L., Shulman G. L., & Corbetta M. (2016). The effects of hemodynamic lag on functional connectivity and behavior after stroke. Journal of Cerebral Blood Flow & Metabolism, 36(12), 2162–2176. 10.1177/0271678x15614846 26661223 PMC5363662

[bib58] Silasi G., & Murphy T. H. (2014). Stroke and the connectome: how connectivity guides therapeutic intervention. Neuron, 83(6), 1354–1368. 10.1016/j.neuron.2014.08.052 25233317

[bib59] Small S. L., Hlustik P., Noll D. C., Genovese C., & Solodkin A. (2002). Cerebellar hemispheric activation ipsilateral to the paretic hand correlates with functional recovery after stroke. Brain: A Journal of Neurology, 125(Pt 7), 1544–1557. 12077004 10.1093/brain/awf148

[bib60] Smith S. M., Beckmann C. F., Andersson J., Auerbach E. J., Bijsterbosch J., Douaud G., … Glasser M. F. (2013). Resting-state fMRI in the human connectome project. NeuroImage, 80, 144–168. 10.1016/j.neuroimage.2013.05.039 23702415 PMC3720828

[bib61] Sporns O., Chialvo D. R., Kaiser M., & Hilgetag C. C. (2004). Organization, development and function of complex brain networks. Trends in Cognitive Sciences, 8(9), 418–425. 10.1016/j.tics.2004.07.008 15350243

[bib62] Tombari D., Loubinoux I., Pariente J., Gerdelat A., Albucher J.-F., Tardy J., … Chollet F. (2004). A longitudinal fMRI study: in recovering and then in clinically stable sub-cortical stroke patients. NeuroImage, 23(3), 827–839. 10.1016/j.neuroimage.2004.07.058 15528083

[bib63] van den Heuvel M. P., & Hulshoff Pol H. E. (2010). Exploring the brain network: a review on resting-state fMRI functional connectivity. European Neuropsychopharmacology, 20(8), 519–534. 10.1016/j.euroneuro.2010.03.008 20471808

[bib64] Van Essen D. C., Smith S. M., Barch D. M., Behrens T. E. J., Yacoub E., Ugurbil K., & WU-Minn HCP Consortium (2013). The WU-minn Human Connectome Project: an overview. NeuroImage, 80, 62–79. 10.1016/j.neuroimage.2013.05.041 23684880 PMC3724347

[bib65] Wang L., Yu C., Chen H., Qin W., He Y., Fan F., … Zhu C. (2010). Dynamic functional reorganization of the motor execution network after stroke. Brain: A Journal of Neurology, 133(Pt 4), 1224–1238. 10.1093/brain/awq043 20354002

[bib66] Wang X., Wong W., Sun R., Chu W. C., & Tong K. Y. (2018). Differentiated effects of robot hand training with and without neural guidance on neuroplasticity patterns in chronic stroke. Frontiers in Neurology, 9 10.3389/fneur.2018.00810 PMC6186842 30349505

[bib67] Ward N. S., & Cohen L. G. (2004). Mechanisms underlying recovery of motor function after stroke. Archives of Neurology, 61(12), 1844–1848. 10.1001/archneur.61.12.1844 15596603 PMC3713312

[bib68] Wei D., Zhuang K., Ai L., Chen Q., Yang W., Liu W., … Qiu J. (2018). Structural and functional brain scans from the cross-sectional Southwest University adult lifespan dataset. Scientific Data, 5(180134). 10.1038/sdata.2018.134 PMC6049036 30015807

[bib69] Wei G.-X., Dong H.-M., Yang Z., Luo J., & Zuo X.-N. (2014). Tai Chi Chuan optimizes the functional organization of the intrinsic human brain architecture in older adults. Frontiers in Aging Neuroscience, 6, 74 10.3389/fnagi.2014.00074 24860494 PMC4029006

[bib70] Wieloch T., & Nikolich K. (2006). Mechanisms of neural plasticity following brain injury. Current Opinion in Neurobiology, 16(3), 258–264. 10.1016/j.conb.2006.05.011 16713245

[bib71] Wong W.-W., Chan S.-T., Tang K.-W., Meng F., & Tong K.-Y. (2013). Neural correlates of motor impairment during motor imagery and motor execution in sub-cortical stroke. Brain Injury, 27(6), 651–663. 10.3109/02699052.2013.771796 23514275

[bib72] Yeo B. T. T., Krienen F. M., Sepulcre J., Sabuncu M. R., Lashkari D., Hollinshead M., … Buckner R. L. (2011). The organization of the human cerebral cortex estimated by intrinsic functional connectivity. Journal of Neurophysiology, 106(3), 1125–1165. 10.1152/jn.00338.2011 21653723 PMC3174820

[bib73] Zeiler S. R., & Krakauer J. W. (2013). The interaction between training and plasticity in the poststroke brain. Current Opinion in Neurology, 26(6), 609–616. 10.1097/WCO.0000000000000025 24136129 PMC4012223

[bib74] Zhao Y., Lambon Ralph M. A., & Halai A. D. (2018). Relating resting-state hemodynamic changes to the variable language profiles in post-stroke aphasia. NeuroImage: Clinical, 20, 611–619. 10.1016/j.nicl.2018.08.022 30186765 PMC6120600

