# Supplementary material for: Synchronization lag in post stroke: relation to motor function and structural connectivity
Source: Netw Neurosci. 2019 Sep 1;3(4):1121–40. doi: 10.1162/netn_a_00105 (PMC6777982; doi:10.1162/netn_a_00105)
Supplement: Supplementary file 1 [file netn-03-1121-s001.pdf]

Wang, X., Seguin, C., Zalesky, A., Wong, W., Chu, W. C., & Tong, R. K. (2019). Supporting information for "Synchronization lag in post-stroke: Relation to motor function and structural connectivity." *Network Neuroscience*, *3*(4), 1121–1140. https://doi.org/10.1162/netn_a_00105

Supplement to

Synchronization lag in post-stroke: Relation to motor function and structural connectivity

Xin Wang[1], Caio Seguin[2], Andrew Zalesky[2,3], Wan-wa Wong[1], Winnie Chiu-wing Chu[4], Raymond Kai-yu Tong[1*]

[1]Department of Biomedical Engineering, The Chinese University of Hong Kong, Hong Kong, China
[2]Melbourne Neuropsychiatry Centre, Department of Psychiatry, University of Melbourne, VIC, Australia
[3]Department of Biomedical Engineering, University of Melbourne, VIC, Australia
[4]Department of Imaging and Interventional Radiology, The Chinese University of Hong Kong, Hong Kong, China

***Corresponding author:**
Raymond Kai-yu Tong
Address: Office Rm 429, Ho Sin Hang Engineering Building, The Chinese University of Hong Kong, Shatin, N.T., Hong Kong
Email: kytong@cuhk.edu.hk
Tel: +852 3943 8454

Supplementary Methods

Image acquisition

Stroke individuals were scanned with a 3T Philips MR scanner (Achieva TX, Philips Medical System, Best, Netherlands) with an 8-channel head coil. The following imaging data were acquired: 1) high resolution T1-weighted anatomical images (TR/TE=7.47/3.45 ms, flip angle=8°, 308 slices, voxel size=0.6×1.042×1.042mm^3) using a T1-TFE sequence (ultrafast spoiled gradient echo pulse sequence); 2) BOLD fMRI images (TR/TE=2000/30 ms, flip angle=70°, 37 slices/volume, voxel size=2.8×2.8×3.5mm^3) using a FE-EPI sequence (gradient-echo echo-planar-imaging sequence); and 3) diffusion-weighted images (TR/TE = 3788/88 ms, flip angle = 90◦, 60 slices, voxel size = 1.5 × 1.5 × 2mm^3) using a diffusion-weighted single-shot spin-echo echo-planar pulse (DWISE) sequence along 32 different diffusion directions with a b-value of 1000 s/mm^2, and an additional baseline (b = 0) image. Individuals were presented with a white crosshair on black background and instructed to rest while focusing on the fixation cross during the fMRI acquisition. The resting state fMRI acquisition lasted for 8 minutes resulting 240 brain volumes.

The imaging datasets of main healthy controls were acquired from an open database[1]. Scanning was performed with 3T MRI scanner (Siemens Trio Tim, Erlangen, Germany) with a 12-channel head coil. High-resolution structural images were acquired using a magnetization-prepared rapid gradient echo (MPRAGE) three-dimensional T1-weighted sequence (TR/TE = 2530/3.39 ms, flip angle = 7◦, FOV = 256 mm, slice thickness = 1.33 mm). Resting state fMRI were obtained using an echo planar imaging (EPI) sequence (TR/TE = 2000/30 ms, flip angle = 90°, 33 slices/volume, voxel size=3.4×3.4×4.0 mm^3) with total of 240 volumes. All of the subjects were instructed to keep their eyes closed and move as little as possible[2]. Diffusion-weighted images were acquired using an EPI sequence (TR/TE = 6600/104 ms, FOV = 230 mm, resolution = 1.8 × 1.8 × 3mm^3) along 64 different diffusion directions with a b-value of 1000 s/mm^2, and an additional baseline (b = 0) image.

Another age-matched healthy control samples were sourced from a recently published database[3]. Twenty age-matched right-handed subjects with no large head motion artifact were selected. The ID of included subjects can be found in Supplementary Table 2. Images were acquired with 3-T Siemens Trio MRI scanner. A magnetization-prepared rapid gradient echo (MPRAGE) sequence was used to acquire high-resolution T1-weighted anatomical images (TR/TE=1900 /2.52 ms, flip angle=90°, slices=176, thickness =1.0 mm, and voxel size=1×1×1 mm^3). The resting-state functional images were obtained using gradient echo echo-planar-imaging (EPI) sequences (TR/TE=2000/30 ms, slices=32, flip angle=90, FOV=220×220 mm, thickness/slice gap=3/1 mm, and voxel size=3.4×3.4×4 mm^3). During the resting-state MRI scan, the subjects were instructed to rest without thinking about anything specific but to refrain from falling asleep. A total of 8 minutes of resting-state data was acquired for each individual.

Resting State Network

Each region in the Desikan/Killiany atlas has been assigned into one of the resting state networks according to a previous study[4], which spatially decomposed the fMRI signal into different independent components using FSL MELODIC. Besides, all the regions in subcortical areas were comprised together to form the subcortical network. Therefore, totally seven subnetworks were delineated in this work.

Supplementary Figures

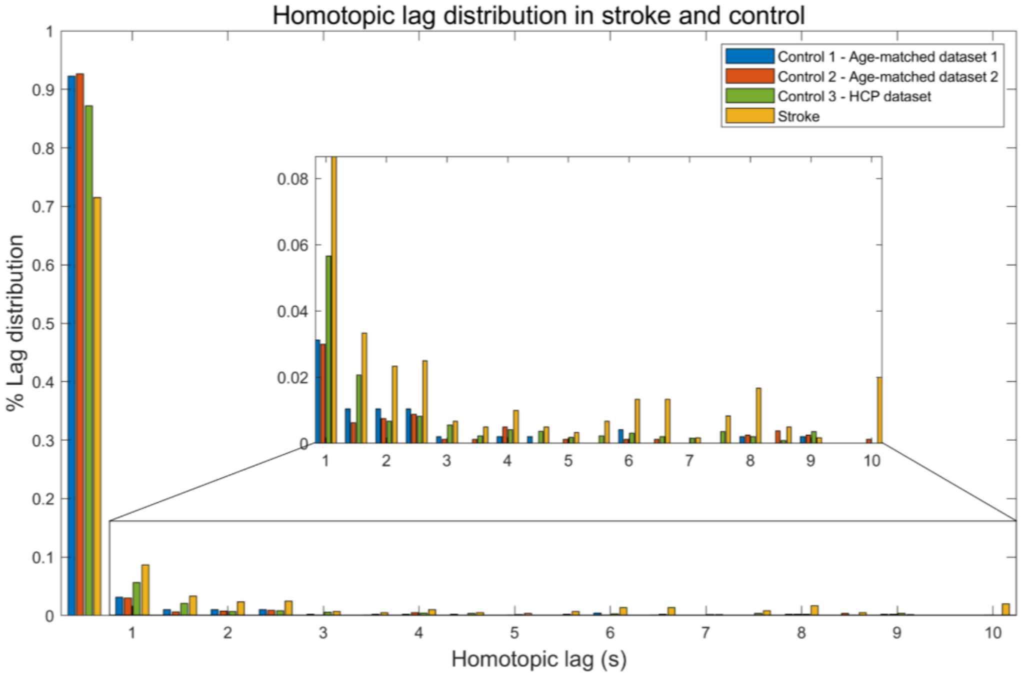

Supplementary Figure 1 Lag distribution between control and stroke. Main text used the age-matched dataset 1 while we also tested the distribution on a larger sample size and samples from a different scanner. To confirm that our results were consistent with different parcellations and TRs, 200 healthy samples sourced from Human Connectome Project (HCP) were analyzed. The brain of HCP data was parcellated into 180 homotopic regions and the TR was 0.72s. The results suggested that stroke individuals did have a substantially longer tail than healthy controls despite the scanners and the TRs.

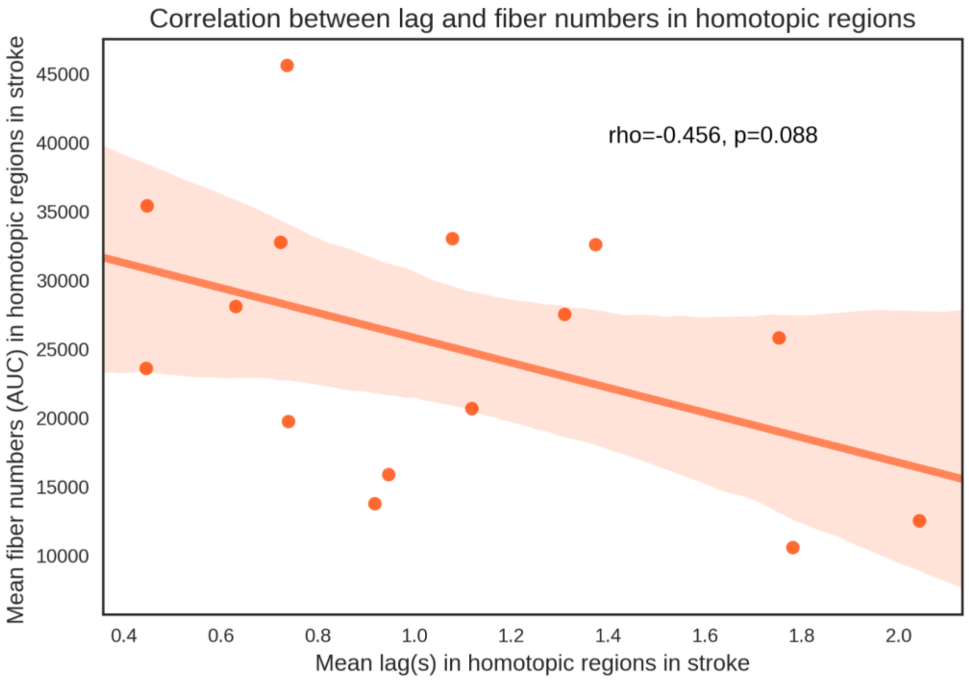

Supplementary Figure 2 Correlation between homotopic lag and fiber numbers across stroke individuals. Lag and fiber numbers were averaged across all homotopic regions for each individual. The fiber numbers were summarized using the area under curve (AUC), computed across structural networks with connection density ranging between 10% and 50%. The relationship implies that fiber numbers have the trend to explain the synchronization lag variation in stroke individuals.

Supplementary Table 1

Correlation between regional lag and distance to the lesion for each individual

ID	r value	P value
S2	1.11E-16	1
S3	-0.101235233	0.534227893
S4	-0.300264291	0.059765676
S5	-0.308852487	0.052487148
S6	-0.348775133	0.027406633*
S7	-0.514158248	0.000689285*
S8	-0.378318884	0.016081993*
S9	-0.35411105	0.024976167*
S10	-0.199210535	0.217813451
S11	-0.134557492	0.407780974
S12	-0.413357324	0.008022189*
S13	-0.295846601	0.063810797
S14	-0.163352327	0.313859874
S15	-0.116465549	0.474191072

Pearson correlation coefficient was used to test the relationship between the lag and the corresponding distance to the lesion for each individual independently. Results indicated that the Euclidean distance to the lesion tended to correlate with synchronization lag in majority of individuals. $P<0.05$ were marked by an asterisk.

Supplementary Table 2

Information of Supplementary Age-matched Control Samples (dataset 2)

ID	Age	Gender
031381	60	F
031382	59	M
031384	60	M
031387	51	F
031388	57	M
031391	57	F
031392	62	M
031393	56	F
031395	61	F
031398	56	M
031399	56	F
031400	62	F
031402	57	M
031407	62	M
031408	58	F
031409	65	F
031410	51	F
031411	62	F
031420	50	M
031431	59	M

Twenty age-matched samples were selected from an open dataset. The selection criteria were: 1) No large head motion artifact; 2) Edinburgh Handedness Inventory score was 100; 3) The gender is relatively evenly distributed. There is no significant difference between these samples and the stroke in age (age-matched dataset 2: 58±4, Stroke: 54±8; p=0.1039).

Reference:

1. IPCAS8. doi:10.15387/fcp_indi.corr.ipcas8
2. Wei, G.-X., Dong, H.-M., Yang, Z., Luo, J. & Zuo, X.-N. Tai Chi Chuan optimizes the functional organization of the intrinsic human brain architecture in older adults. *Front. Aging Neurosci.* **6**, 74 (2014).
3. Wei, D. *et al.* Structural and functional brain scans from the cross-sectional Southwest University adult lifespan dataset. *Sci. Data* **5**, 180134 (2018).
4. Schirner, M., McIntosh, A. R., Jirsa, V., Deco, G. & Ritter, P. Inferring multi-scale neural mechanisms with brain network modelling. *eLife* **7**, (2018).
